# Decolonizing medical education: a systematic review of educational language barriers in countries using foreign languages for instruction

**DOI:** 10.1186/s12909-025-07251-2

**Published:** 2025-05-13

**Authors:** Abdullah Ashraf Hamad, Doaa B. Mustaffa, Asmaa Zakria Alnajjar, Raghad Amro, Mohammad Ghassab Deameh, Bassant Amin, Ibraheem M. Alkhawaldeh

**Affiliations:** 1 Faculty of Medicine, Menoufia University, Menoufia, Egypt; 2 Faculty of Medicine, Alexandria University, Alexandria, Egypt; 3 Faculty of Medicine, Al-Azhar university, Gaza, Palestine; 4 Faculty of Medicine, Mutah University, Al Karak, Jordan; 5 Prince Hamza Hospital, Amman, Jordan

**Keywords:** Medical education, Language of instruction, Language barriers, English, Native Language, Mother tongue

## Abstract

**Background:**

Language barriers in medical education, particularly in countries where foreign languages are used as the medium of instruction, pose significant challenges for domestic medical students. These barriers hinder academic performance, comprehension, and communication with patients, ultimately impacting the quality of healthcare delivery. Despite the prevalence of this issue, a comprehensive understanding of its effects remains underexplored. This systematic review aims to synthesize evidence on language barriers in medical education and propose strategies to address them.

**Methods:**

Following PRISMA guidelines, we conducted a systematic review of studies published up to March 21, 2024, using PubMed, Scopus, and Web of Science. Eligible studies focused on language barriers faced by medical, pharmacy, nursing, dental, or veterinary students in countries relying on foreign-language-based medical education. Data extraction included study characteristics, reported language barriers, and their impact on education and patient communication. Quality assessment was performed using the Mixed Methods Appraisal Tool.

**Results:**

From 5,410 citations, 49 studies involving over 14,500 students met the inclusion criteria. Most studies (*n* = 32) were conducted in Arab countries, with 15 in Saudi Arabia. Two key themes emerged: (1) Education and Academic Performance: Students frequently reported difficulties comprehending foreign-language textbooks, lectures, and assessments, leading to poor academic outcomes, increased stress, and higher dropout rates. (2) Communication Skills with Patients: Studying and training in a foreign language hindered students’ ability to communicate effectively with patients in their native language, impacting empathy, medical history collection, and overall patient care. Many studies highlighted students felt more confident and effective when using their native language during clinical interactions.

**Conclusion:**

Language barriers in foreign-language-based medical education significantly impede students’ academic performance and patient communication skills. Addressing these challenges through reforms, such as integrating native language instruction and supplemental language training, is crucial to enhancing medical education quality and ensuring effective healthcare delivery. Future research should explore innovative solutions, including bilingual education and AI-driven translation tools, to bridge these gaps.

**Supplementary Information:**

The online version contains supplementary material available at 10.1186/s12909-025-07251-2.

## Introduction

Medicine is a cornerstone of public health and individual well-being. Quality medical education plays a pivotal role in shaping competent healthcare professionals who are not only technically skilled but also empathetic and patient-centered [1, 2]. By equipping future doctors, nurses, and other healthcare providers with essential knowledge and skills, medical education lays the foundation for effective diagnostics, treatment, and patient care. Thus, ensuring that medical education is both effective and accessible is critical to strengthening healthcare systems globally [2].

Language is a key factor in the effectiveness of medical education. As the primary medium of instruction, language shapes how medical knowledge is conveyed—whether through textbooks, lectures, or clinical interactions with patients [3, 4]. It is a vital learning tool that facilitates comprehension, academic performance, and professional communication. Therefore, the choice of language in medical education profoundly influences the quality of learning outcomes and the preparedness of healthcare providers [3].

**Fig. 1:**
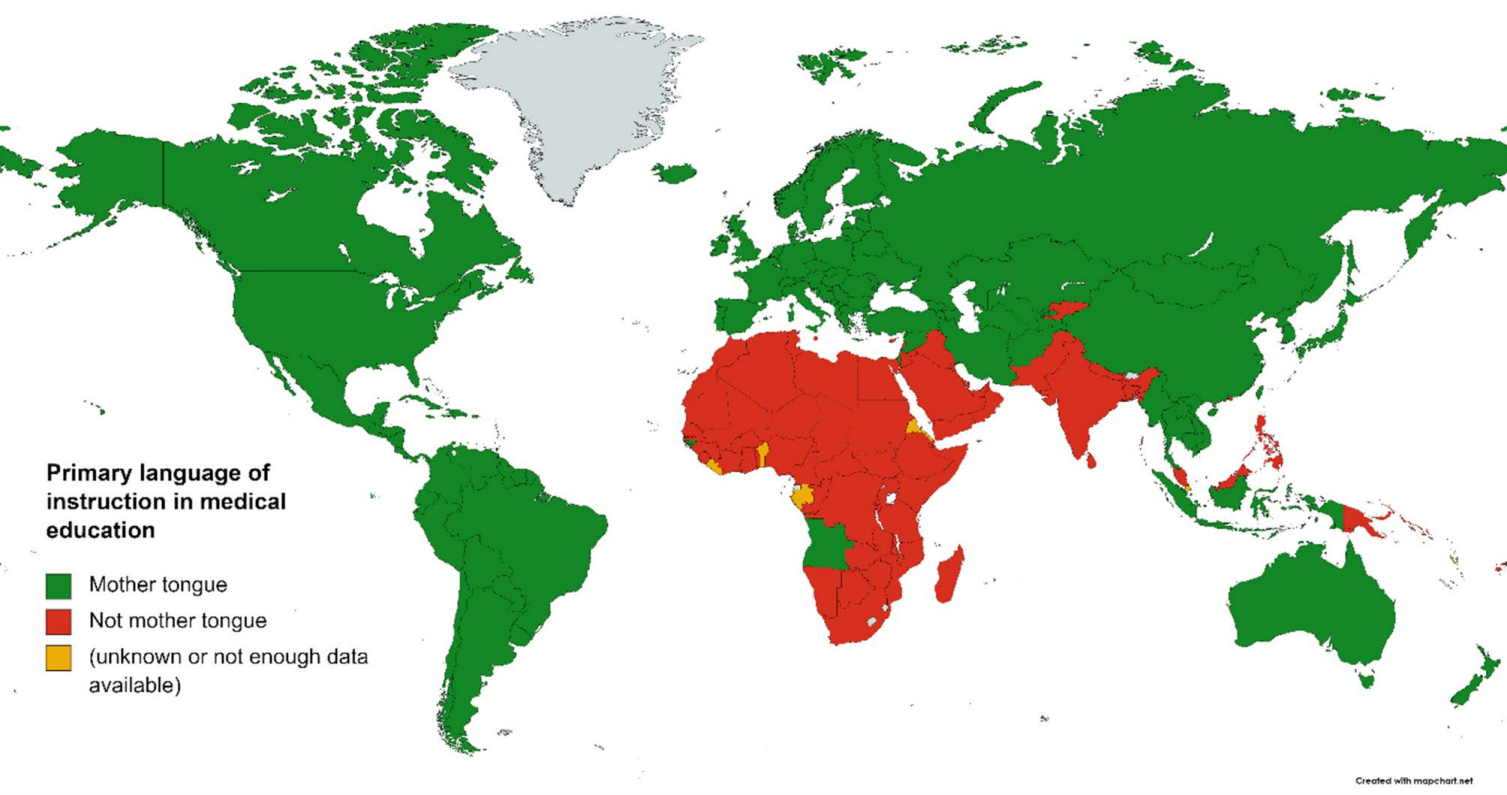
The status of reliance on native languages in medical education worldwide. (Adapted from Hamad A.A., 2023; doi: 10.1016/j.glmedi.2023.100007)

A global screening of instructional languages in medical education revealed that 105 countries use their native languages as the primary medium of instruction. Conversely, 74 countries—primarily in developing regions, including Africa and the Arab world—rely on foreign languages for medical education (Fig. 1) [5]. This reliance often stems from historical legacies, such as colonialism, which imposed foreign languages on educational systems [5, 6]. While adopting a foreign language (mostly English) can offer access to extensive global medical knowledge, it may also create significant challenges for domestic students [3, 7–9]. Research highlights that education in a native language fosters better comprehension, academic performance, and communication, all of which are critical for effective patient care [3].

Reliance on a foreign language in medical education introduces numerous barriers. Students may struggle to grasp complex medical concepts, leading to gaps in understanding and lower academic performance [7, 10]. Furthermore, their ability to communicate effectively with patients in their native language may be hindered, impacting the quality of care [11]. This language barrier not only affects learning but also disrupts the critical connection between medical professionals and their patients.

Given these challenges, it is essential to examine the impact of using foreign languages in medical education on students’ learning experiences, communication skills, and, ultimately, the quality of healthcare delivery. This study aims to systematically review the challenges faced by domestic medical students in countries that adopt a foreign language as the primary medium of instruction in medical education.

## Methods

### Search strategy

This systematic review adhered to Preferred Reporting Items for Systematic Reviews and Meta-Analyses (PRISMA) [12]. A comprehensive search was conducted across PubMed, Scopus, and Web of Science databases up to March 21, 2024. The search terms included: (“medical education” OR “medical students” OR “nursing students” OR “pharmacy students” OR “dental students” OR “dentistry students” OR “veterinary students”) AND (language or linguistic) AND (barriers OR difficulties OR challenges OR obstacles OR problems OR issues OR struggles OR dilemmas OR limitations). No filters or language restrictions were applied. Additionally, the references of the included studies were screened to ensure comprehensive coverage.

### Inclusion criteria

This review aimed to summarize language barriers encountered by medical students studying in countries where foreign languages are integral to medical education. The inclusion criteria were as follows: (a) primary studies involving medical, pharmacy, dental, veterinary, or nursing students; (b) studies conducted in countries relying on foreign languages in medical education [5]; and (c) studies reporting language barriers experienced by students in various educational aspects (e.g., comprehension, patient communication). Exclusion criteria included studies focusing on: (a) language barriers faced by international students studying abroad; (b) language barriers affecting specific minority groups within a country; (c) faculty or healthcare professionals without student involvement; (d) language barriers experienced by patients. Reviews, editorials, and abstracts were also excluded.

### Outcomes of interest

This review concentrated on two primary outcomes. Firstly, it examined language barriers encountered by students within the educational context, encompassing comprehension of concepts, study time, information retention, and participation in discussions. Secondly, the review explored language barriers during student-patient communication in training or Objective Structured Clinical Examinations (OSCEs) exams. Specifically, it aimed to analyze how students communicate with patients in their native language following education in a foreign language.

### Screening, data extraction, and quality assessment

Two reviewers independently screened the titles and abstracts of the identified citations. Full texts of relevant articles were then reviewed by a third author for final inclusion decisions. Data extraction was carried out by two authors using an online form, covering study year and country, design and sample size, participants, objectives, main findings, and reported language barriers. Quality assessment was conducted by two reviewers utilizing the Mixed Methods Appraisal Tool to evaluate the various study designs (qualitative, quantitative, and mixed methods) [13]. Any discrepancies during screening, data extraction, or quality assessment were resolved through discussion with a third author.

### Analysis

Qualitative synthesis followed the methodology for narrative reviews outlined in the Cochrane Handbook [14]. Language barriers were categorized into two themes: (1) Educational and Academic Performance Barriers and (2) Patient Communication Barriers. A summary table was created to systematically present extracted data, including study ID, country, study design, participants, sample size, proportion of male participants, study aims, key findings, and reported language barriers. This approach enabled structured comparison and exploration of findings across diverse contexts and study designs. Additionally, the quality of the included studies was analysed and summarized in a table following the criteria outlined above.

## Results

### Characteristics of the included studies

**Fig. 2:**
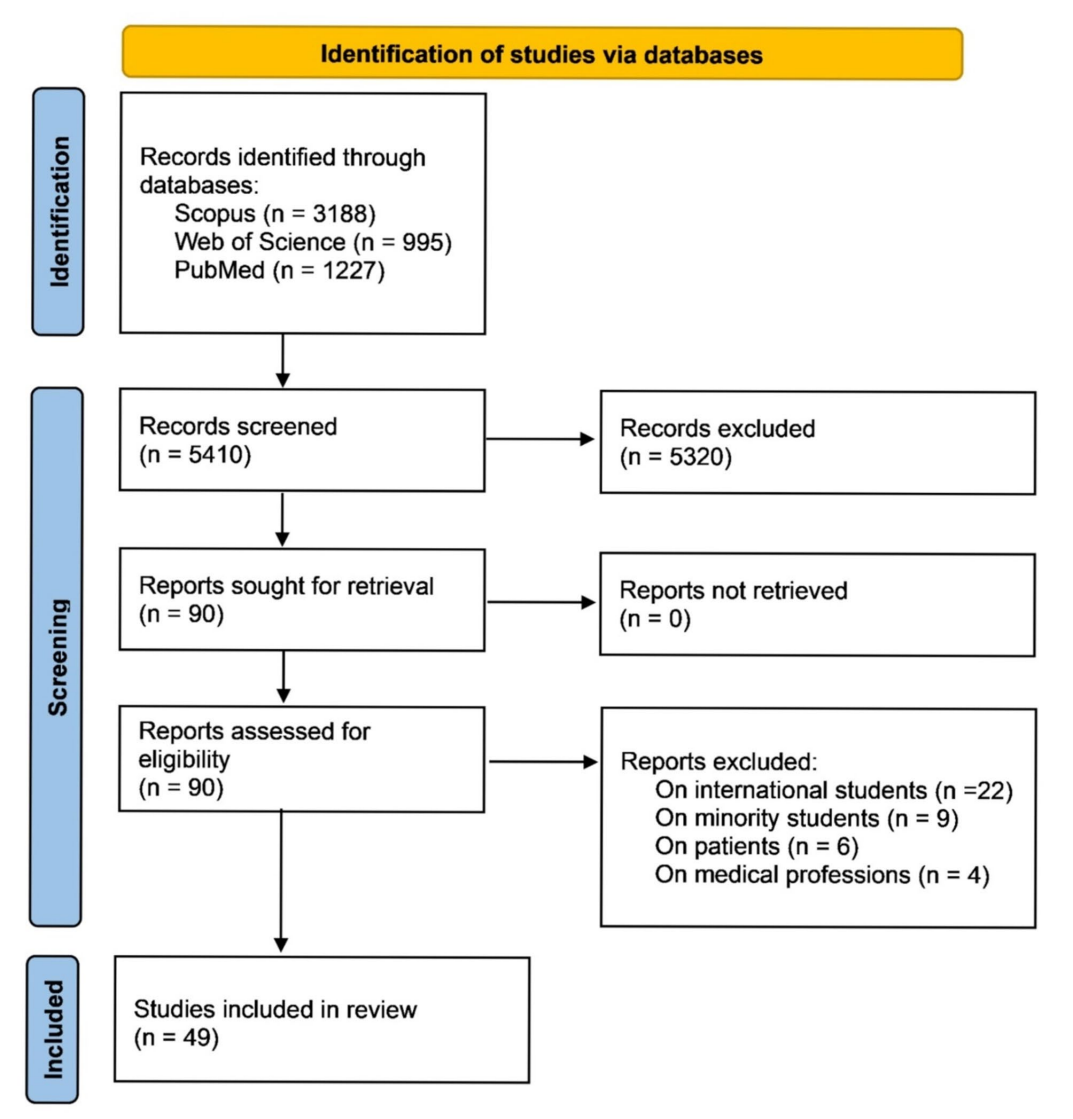
The PRISMA flow diagram

Our search yielded a total of 5,410 citations. After screening titles and abstracts, we identified 90 records for eligibility assessment. Among these, 22 studies focused on international students studying in foreign countries, four on medical professionals, six on patients, and nine on students from minority groups (Fig. 2). Ultimately, 49 studies involving over 14,500 students met the inclusion criteria and were included in our analysis [4, 7, 8, 10, 11, 15–59]. All studies were conducted in countries that utilize second-language-based medical education. The majority (*n* = 32) were carried out in Arab countries, with 15 studies conducted in Saudi Arabia. The studies comprised seven qualitative, six mixed-method, and the remainder quantitative research. Two studies were published in Arabic, while the others were in English. Participants primarily consisted of medical students, although some studies also included pharmacy, nursing, dental, and veterinary students. The overall quality of the studies was generally good, as detailed in the Supplementary file. Table 1 summarizes the characteristics, aims, and main findings of the included studies.

**Table 1:** Characteristics, main findings, and language barriers reported in the included studies

Study ID	Country	Study design	Participants	Sample size	Males, *n* (%)	Aim	Findings	Language barriers reported
Abdulghani 2014	Saudi Arabia	Qualitative focus group discussions-based study	High academic achieving medical students from the second, third, fourth and fifth academic years	19	10 (53)	To explore the high achieving students’ perceptions of factors contributing to academic achievement	Key factors for high academic achievement were lecture attendance, early revision, prioritizing learning, deep learning, group study, mind mapping, skills lab practice, patient interaction, learning from mistakes, time management, and family support.	Studying in a foreign language was defined as a negative factor to academic achievement.
Abi Raad 2016	Lebanon	Quantitative survey-based study	Medical students rotating clinical clerkship	457	241 (53)	To investigates the association between medical education in a foreign language and students’ confidence in their history-taking skills in their native language	The majority of students (88.5%) reported being confident in conducting a medical history in their native language.	One third of students perceived that education and training in a foreign language may have negatively affected their communication in Arabic.
Abou Sahda 2021	Morocco	Quantitative survey-based study	Medical students in different school years	891	363 (41)	To investigate the attitudes of medical students regarding their learning challenges and expectations in relation to linguistic reform initiatives at universities	A third of respondents said French hindered their success. Over half saw links between language, comprehension, and identity. Most favored switching to English (69%) or partial (42%) or total (37%) Arabization of medical courses.	A third of respondents acknowledged that the use of French hindered their academic success, citing increased learning time (44%) and reduced ability to communicate with patients (21%).
Ahmed 1988	Kuwait	Mixed methods	Medical students	42	-	To investigate the factors influencing the performance of medical students at the Faculty of Medicine, University of Kuwait	Results showed strong correlations between high school grades and medical performance, along with English proficiency. However, controlling for English proficiency weakened the relationship between high school GPA and clinical GPA.	English language proficiency (the foreign language of instruction) influenced academic achievement.
Alhamami 2021	Saudi Arabia	Quantitative survey and record-based study	Medical students and instructors	3536	-	To examine the impact of using English as the language of instruction and analyze the experiences of students and instructors in a healthcare course in Saudi Arabia	Data from the past five years showed first-semester English grades predict final GPAs. Both students and instructors agree that using English in healthcare courses creates challenges, especially for students with limited fluency, impacting their performance.	Questionnaire results showed that both students and instructors find using English in healthcare subjects challenging, especially for students with limited fluency, impacting their academic performance.
Al-Mahmoud 2013	Saudi Arabia	Quantitative survey-based study	Nursing students	498	121 (24)	To explore the motivations of Saudi nationals for pursuing nurse training and the appeal of nursing as a career for them	Over 60% chose nursing as their first choice, with most motivated by a desire to become health professionals. Many agreed that nursing is viewed negatively in Saudi society.	Many students cited heavy workloads, theoretical studies, and the challenges of studying in English as significant factors contributing to dropout rates.
Almoallim 2010	Saudi Arabia	Quantitative survey-based study	Second-year medical and medical sciences students	270	128 (47)	To investigate the difficulties encountered by first-year medical students	Students ranked peer competition as the top difficulty, followed by poor English skills. Males identified peer competition as their top concern, while females ranked it fourth. Most were dissatisfied with passive teaching and sought improvements in English.	Poor English proficiency (the language of instruction) was ranked second overall and first among female students as a difficulty facing them.
Alnahdi 2021	Saudi Arabia	Quantitative survey-based study	Medical students from the fourth to the sixth year	290	205 (71)	To identify the effects of learning history-taking in English on its application in medical students’ native language (Arabic)	Of the students, 33.8% felt confident taking histories in Arabic, 47.6% preferred OSCE training in Arabic, and the mean difficulty level was 2.1 ± 0.7. Additionally, 68% recommended adding short Arabic history courses.	Studying in a foreign language only made one-third of students feel confident taking histories in their native language.
Alqahtani 2022	Saudi Arabia	Quantitative survey-based study	Nursing students from different years	90	45 (50)	To identify the level of English language usage and its effect on academic achievement among Saudi undergraduate nursing students	Saudi nursing students had high academic achievement but low English language usage. Their highest average score was in listening, followed by reading, writing, and speaking. English language usage significantly influenced academic achievement.	English language usage (the foreign language of instruction) influenced academic achievement.
Ameayou 2023	Morocco	Quantitative survey-based study	Medical and pharmacy students	450	-	To assess students’ understanding of French language training, challenges in patient communication, and their acceptance of medical studies in Arabic	16% of students had trouble with French lessons, and 48.9% struggled to communicate with patients. Only 22% were willing to study medicine in Arabic. Regarding Arabic instruction’s impact, 42.2% saw a positive effect on training, 85.2% on patient communication, and 64.8% on care quality.	Overall, studying in French (the foreign language of instruction) was challenging, leading to a preference for studying in the native language.
Amulya 2021	India	Quantitative survey-based study	Medical students from different years	165	75 (46)	To assess medical students’ perceptions of the impact of language barriers on bedside teaching and learning	Only 11.5% of students could communicate comfortably in the local language, while 88.5% could not.	Studying in a language different from the local language created barriers to academic success and communication. Most students supported conducting classes exclusively in the local language during the first year.
Ariyasinghe 2013	Sri Lanka	Quantitative survey-based study	Dental student	306	111 (36)	To investigate the predictors of academic performance among first-year dental undergraduates	The analysis revealed that English language proficiency, gender, and prior academic ability were significant predictors of GPA.	English language proficiency (the foreign language of instruction) significantly influenced academic achievement.
Chhabra 2022	Mauritius	Qualitative interviews-based study	Final-year medical students	12	-	To explore medical students’ perspectives on the factors influencing empathy development during their undergraduate training	Medical students reported improved empathy, linked to patient interactions, positive role models, and personal growth. Barriers included exams, overload, stress, negative role models, and lack of formal empathy training.	Some students identified the local language (different from the instructional language) as a barrier to showing empathy. They suggested including the regional language as a special module in the official curriculum.
Diab 2019	Qatar	Quantitative experimental study	Pharmacy students	22	0 (0)	To assess student performance and perceptions after completing two Objective Structured Clinical Examinations (OSCEs), one in English and the other in their native language, Arabic	Overall scores were similar, but student rankings differed. Students felt more confident performing in Arabic, viewed the Arabic exam as more reflective of real practice, and believed that using Arabic OSCEs could improve patient care.	Students felt more confident in their native language (Arabic), found the Arabic exam more reflective of real practice, and believed that Arabic OSCEs could enhance patient care.
Eagleton 2015	South Africa	Mixed methods survey-based study	Anatomy and physiology students	76	-	To explore the factors contributing to learning satisfaction among first-year anatomy and physiology students	Students cited distractions that hindered their focus and preferred seeking help from peers over lecturers. Studying in a second language was a key barrier to success. Opinions varied on the use of technology for learning anatomy and physiology.	Most students studied in their second language, which was identified as a barrier to success.
Gazzaz 2023	Saudi Arabia	Quantitative survey-based study	preparatory year medical students	507	233 (46)	To compare the performance of Arabic-speaking students on a diabetic questionnaire presented in Arabic (their native language) with their performance on the same questionnaire in English	Students scored higher in knowledge and attitudes on the Arabic questionnaire compared to the English version. Females had higher knowledge scores in both versions. Regression analysis showed that students performed better on the Arabic questionnaire.	Students performed significantly better in their native language than in English.
Hasan 2017	United Arab Emirates	Quantitative experimental study	Third-year bachelor of pharmacy students	72	-	To assess student communication and patient management skills through the introduction of Arabic and simulated patient assessments in a communication and counseling course	Students achieved similar scores in assessments across the course, regardless of whether they were in the Arabic or English groups. They positively evaluated the course changes and offered constructive feedback on the usefulness and adequacy of the content.	Most students agreed that the Arabic material in the course enhanced their communication skills (85%), was adequate for assessment preparation (70%), and better prepared them for future practice (73%).
Hassan 1995	Oman	Quantitative cross-sectional	Medical students	84		To examine the effect of English language courses on medical students’ academic performance	Performance in all disciplines of the alimentary system exam showed a significant positive correlation with English, similar to the strong correlation found in foundation sciences, zoology, chemistry, and physics.	English proficiency, the foreign language of instruction, emerged as a key determinant affecting academic outcomes.
Hashim 2013	United Arab Emirates	Quantitative experimental study	Third-year medical students	45	10 (22)	To determine whether Arabic-speaking medical students faced difficulties with the various components of communication skills training conducted in English	Tutors assigned the lowest marks for students’ abilities to express empathy, inquire about patients’ feelings, use transition statements, assess functional impact, and elicit patients’ expectations (*P* < 0.001).	Learning communication skills in a foreign language was associated with difficulties in complex communication, particularly in expressing empathy and eliciting patients’ expectations and feelings.
Higgins-Opitz 2012	South Africa	Quantitative survey-based study	Second-year medical students	324	115 (35)	To examine student perceptions of the Active Physiology Learning exercise concerning three parameters: sex, language, and self-reported performance in class tests	Student responses varied significantly for 27 of the 50 questionnaire items based on sex (22%), home language (37%), performance (26%), and their combinations. Results confirmed that sex, home language, and performance significantly influence student motivation to learn.	Weakness in English (the foreign language of instruction) was a key factor affecting academic outcomes.
Ismaiel 2023	United Arab Emirates	Quantitative experimental study	Medical students	59	18 (31)	To describe the development, implementation, and initial perceptions of a native language program for medical students	Early perceptions of the program were positive, with 89.6% of students using the information during home visits and clinical rotations, and 87.5% feeling more comfortable communicating with Arabic-speaking patients.	A native language-based program was more acceptable, comfortable, and yielded better outcomes.
Jabali 2022	Palestine	Mixed methods study	Medical students	604	194 (32)	to investigate medical students’ perceptions and attitudes toward the language(s) of instruction at two Palestinian universities	The study reveals a divide among students on the preferred language of medical instruction: some favor Arabic, while others are comfortable with English terminology. Significant differences were noted based on gender, major, and year of study.	Half of the students preferred Arabic over English as the language of instruction. Many interviewees expressed that using English might hinder their ability to communicate with patients after graduation.
Jameel 2019	Saudi Arabia	Quantitative survey-based study	Medical students	347	123 (36)	To examine medical students’ preferences for learning resources and their study habits at university	Female students spent more time reading textbooks than males, who preferred lecture handouts. One-third struggled with textbooks due to limited English, leading 19.3% to lose interest. Males watched twice as much television as females.	Approximately one-third of students (34.3%) reported difficulty understanding English-language textbooks due to limited English proficiency, while 19.3% expressed a lack of interest in these materials.
Jha 2019	India	Quantitative survey-based study	Medical students	90		To examine the role of language proficiency and personality traits on the academic performance of undergraduate medical students	Analysis showed that English language proficiency significantly influenced academic performance. High-performing students mostly exhibited an internal locus of control and moderately high self-evaluation.	English proficiency, the foreign language of instruction, emerged as a key determinant of academic performance among medical students.
Kaliyadan 2015	Saudi Arabia	Quantitative cross-sectional	Preparatory year medical students	103	49 (48)	To correlate English language proficiency with academic performance among medical students in their preparatory year	A significant positive correlation was found between English exam scores and both written and oral exam scores in the medical examination. However, no significant correlation was observed with other components, such as assignments, presentations, and portfolios.	English proficiency, the foreign language of instruction, emerged as a key determinant of academic performance among medical students.
Khallof 2019	Saudi Arabia	Quantitative survey-based study	Dental students	378	-	To explore the language difficulties faced by Arabic dental students and dentists in their education and assess their attitudes toward Arabizing the medical curriculum	70% of respondents felt that studying in Arabic is essential for Arabs, with a similar number agreeing that mastering their native language is easier than learning another. Over 65% preferred a mix of both languages for lectures and exams.	Students unanimously agreed on the necessity of receiving knowledge in Arabic and emphasized the importance of studying their mother tongue to improve understanding and memorization.
Khan 2004	Oman	Qualitative survey-based study	Pharmacy students	39	-	To examine challenges in pharmacy education and English language instruction, assess the relevance of technology integration as a blended learning strategy, and evaluate the feasibility of suitable teaching materials	The use of English as the language of instruction was identified as a significant barrier to learning pharmacy-related subjects, with many students finding pharmacy textbooks challenging. The study also highlighted that integrating technology could be beneficial.	English proficiency, the foreign language of instruction, emerged as a key determinant of academic performance among medical students.
Khan 2019	Saudi Arabia	Qualitative focus group discussions-based study	Medical students	8		To investigate the oral communication barriers faced by Arab medical students	Medical students frequently engaged in oral interactions with peers and professors, relying on strong oral communication skills for success. They encountered both internal and external barriers to effective communication.	One fourth of the students were found to think of English as an alien language. English proficiency affected the performance in the oral exams.
Khan 2021	Saudi Arabia	Mixed methods study	Pharmacy students	39		To explore issues in pharmacy education and specific English, investigate the relevance of technology integration as a blended learning strategy, and assess the feasibility of suitable teaching materials	English as the language of instruction was identified as a significant barrier to learning pharmacy-related subjects, with many students finding pharmacy textbooks challenging. However, the study indicated that technology integration could be beneficial.	English as the foreign language of instruction was identified as a significant barrier to learning pharmacy-related subjects.
Lucas 1997	Hong Kong	Quantitative cross-sectional	Medical students	82		To examine how language acts as a barrier to acquiring anatomical knowledge among medical students	The study found a strong correlation between students’ entrance levels in English and their final exam results, as well as a significant relationship between their English proficiency and class test scores.	English proficiency, the foreign language of instruction, emerged as a key determinant of academic performance among medical students.
Matthews 2018	South Africa	Qualitative focus group discussions-based study	Medical students, educators and stakeholders	24		To explore the relationship between communication training and social accountability at a single institution	Participants valued good communication, but it was often poorly role-modeled. They felt communication and native language instruction lacked support to meet community needs. Students had limited understanding of social accountability.	Studying in English while neglecting the native language impacted students’ communication skills.
McLean 2013	United Arab Emirates	Qualitative survey-based study	First-year medical students	139	40 (29)	To examine how poor English language proficiency hinders the development of generic skills among first-year medical students	Students noted improvements in information-handling and communication skills due to medical communication activities. However, they felt that poor English skills hindered their abilities and negatively affected classroom participation and interactions with teachers.	English proficiency, the foreign language of instruction, emerged as a key determinant of academic performance, classroom participation and interactions with teachers.
Mirza 2010	United Arab Emirates	Quantitative survey-based study	Third-year medical students	36	12 (33)	To investigate how learning communication skills in English impacts non-English speaking medical students	Nearly 72% of students felt confident taking a history in English, while only 28% felt confident doing so in Arabic. Half expected to communicate primarily in Arabic after training, with only 8% anticipating using English.	Less than a third of students felt confident taking patient history in their native language after learning communication skills in English.
Mpofu 1998	United Arab Emirates	Quantitative survey-based study	Preparatory year medical students	49	18 (37)	To examine the impact of English proficiency on problem-based learning interactions among preparatory year medical students	Students’ participation in problem-based learning sessions strongly correlated with TOEFL scores. Female students mainly used English, while males often switched to Arabic. TOEFL scores were the best predictor of participation.	English proficiency, the foreign language of instruction, emerged as a key determinant of problem-based learning performance.
Oducado 2020	Philippines	Quantitative retrospective descriptive study	Nursing graduates	141		To examine the relationship between English language proficiency, academic performance, and success in the Nurse Licensure Examination	The study found significant correlations between academic performance, Verbal Ability scores, and English courses in the nursing curriculum. Verbal Ability and English course performance were also significantly linked to Nurse Licensure Examination ratings.	English language proficiency, the foreign language of instruction, was a key factor in determining both academic performance and licensure success among nursing students.
Olajuyin 2022	Nigeria	Quantitative survey-based study	Medical students	312	176 (56)	To explore Nigerian medical students’ views and practices regarding the use of Yorùbá (an indigenous language) during their clinical clerkship	Most students (70.8%) used Yorùbá during clinical clerkship, despite being taught in English. The majority (73.7%) supported adding indigenous language training to the medical curriculum to enhance communication skills.	The majority of students (73.7%) supported incorporating indigenous language training into the medical curriculum, believing it would improve communication skills.
Phisalprapa 2016	Thailand	Quantitative cross-sectional	Four-year medical students	295		To investigate the impact of English-language multiple-choice test questions on the test scores of medical students	The mean MCQ scores in Thai (65.0%) were significantly higher than in English (56.5%). Only 24.7% of students scored higher on English tests than on Thai ones. MCQ scores in Thai correlated more closely with total course grades than English scores.	The use of English, as the foreign language of instruction, in multiple-choice question tests led to a decrease in student scores.
Pun 2023	Hong Kong	Quantitative survey-based study	Veterinary Medical Students	122	67 (55)	To explore students’ perceptions of sustainable disciplinary language learning in an English-medium instruction university	Student veterinarians were trained for demanding clinical duties, such as diagnosing and writing reports. However, the English-medium instruction posed challenges that hindered their learning effectiveness, revealing a gap between academic tasks and workplace requirements.	The English-medium instruction, being a foreign language, created challenges that impeded students’ learning effectiveness.
Qadeer 2023	Saudi Arabia	Mixed method	Pre-clinical medical students	67	67 (100)	To investigate pre-clinical medical students’ perceptions of their difficulties with English language proficiency at King Khalid University in Saudi Arabia	The findings showed that students faced significant challenges in English language skills, with writing (27.95%) being the most difficult, followed by reading (25.36%), speaking (24.86%), and listening (12.73%).	Medical students reported significant difficulties with English, the foreign language of instruction, which negatively affects their academic achievement.
Rabadi 2020	Jordan	Mixed method	First-year medical students	684	259 (38)	To examine English as a Foreign Language writing anxiety among medical students, focusing on its levels, types, and causes	The results indicate that participants experience high writing anxiety, primarily cognitive anxiety. Key causes include linguistic difficulties, insufficient writing practice, low self-confidence, and fear of writing tests. Qualitative data from semi-structured interviews support these findings.	The results indicated that participants experience high writing anxiety in English, the foreign language of instruction, with cognitive anxiety being the primary type.
Sabbour 2012	Egypt	Quantitative survey-based study	Medical students and staff	550	163 (30)	To examine language barriers in medical education and attitudes toward the Arabization of medicine among students and staff	Most students (56.3%) did not see learning medicine in English as a barrier, but 44.5% of staff did, primarily in the first year. Additionally, 44.8% of students translated English terms into Arabic, and 70.6% preferred learning patient history-taking in Arabic during clinical years.	Many students encounter language barriers in their education, with nearly half needing to translate terms into their native language.
Seneviratne 2019	Sri Lanka	Quantitative survey-based study	Medical students from different years	837	327 (39)	To evaluate medical students’ perceptions of the medium of instruction in their education	More than half of the students (53.4%) believed that having medical textbooks in Sinhala (native language) would enhance their understanding of concepts, and nearly two-thirds (61%) felt their clinical examination performance would improve if conducted in Sinhala.	The use of English as a foreign language of instruction negatively affected academic performance, with most students believing that switching to their native language would improve understanding and performance.
Sheikh 2022	Saudi Arabia	Qualitative focus group discussion-based study	Medical students from different years	22	13 (59)	To examine medical students’ perceptions of language barriers in clinical teaching and learning	Four main themes emerged: challenges in patient interaction, clinical skills development, managing language barriers, and student recommendations. Participants suggested improving Arabic language courses and adding a professional skills course in Arabic for non-native students.	The use of English as a foreign language of instruction negatively affected academic performance and communication skills with patients and students suggested adopting their native language in education and practice.
Shukaili 2023	Pakistan	Quantitative survey-based study	Nursing students	101	80 (79)	To examine the challenges and barriers to providing compassionate care among undergraduate nursing students	Key challenges include inconsistent workloads, neglect of nurses’ needs, lack of compassionate role models, routine-focused care, gender bias, absence of holistic care, negative attitudes, superficial friendliness, and language barriers.	English, the foreign language of instruction, was identified as a significant barrier to providing compassionate care by 65.3% of students.
Singh 2011	India	Quantitative survey-based study	Medical students	336	212 (63)	To examine the prevalence of depressive symptoms and their relationships with socio-demographic variables among medical students at a private medical college	Half of students reported depressive symptoms, particularly in first-year (59.3%) and second-year (65.6%) students, compared to third (34.4%) and fourth-year (37.2%) students. Associated factors included substance abuse, being in early years, and female gender.	Receiving pre-university education in a language other than English was a significant factor associated with the development of depressive symptoms.
Stupart 2008	South Africa	Quantitative analysis study	Final-year medical students	604	235 (39)	to examine the factors affecting oral and clinical surgery examinations among final-year medical students.	Students speaking English at home outperformed others in all exams. Females scored slightly higher overall than males but showed similar performance in clinical and oral exams. Significant score disparities existed among demographic groups.	English, the foreign language of instruction, was identified as a factor influencing exam performance among students who do not speak it as their home language.
Tantawi 2016	Saudi Arabia	Quantitative cross-sectional	First-year dental students	89	44 (49)	To evaluate the scientific writing skills of students completing their preparatory year in a Bachelor of Dentistry program	Female students used significantly more words than male students, whose assignments had lower Flesch reading ease scores. Male students were less likely to use references (OR 0.04) and more likely to make punctuation (OR 2.63) and grammar (OR 3.91) errors.	Writing skills in English, the foreign language of instruction, were insufficient among dental students.
Tayem 2020	Bahrain	Quantitative survey-based study	Final-year medical students	99	37 (37)	To examine the perceptions of final-year Arab medical students regarding language barriers and their impact on learning and academic performance, as well as their language preferences for medical education	Most students did not see a language barrier, regardless of English proficiency (*P* = 0.088). Although most felt language issues didn’t complicate their studies, proficiency made a difference (*P* = 0.005). While 82% were unfamiliar with Arabic medical terms, 66% were confident in communicating with patients in Arabic.	Over a third of students (36%) favored teaching in both Arabic and English. Most did not find language a barrier to studying, though those with lower English proficiency felt it was more challenging (*P* = 0.005).
Tenney 2019	Hong Kong	Quantitative cross-sectional	Pharmacy students	113	58 (51)	To investigate the relationship between English language proficiency and graduating GPA among pharmacy students	A stronger correlation was found between pre-admission English proficiency scores and graduating GPA compared to math, chemistry, or Chinese language scores, with English proficiency being the strongest predictor regardless of gender.	English language proficiency, the foreign language of instruction, was a key factor in determining both academic performance (GPA).

Abbreviations: *OSCE* Objective Structured Clinical Examination, *GPA* Grade Point Average, *MCQ* Multiple Choice Question, *TOEFL* Test of English as a Foreign Language

### Barriers in education and academic performance

Numerous studies have highlighted significant barriers in education that adversely affect academic performance among medical and nursing students (Table 1). A recurrent theme is the challenge posed by language proficiency, particularly in English as the foreign language of instruction. For instance, studies indicated that students often struggle with understanding English-language textbooks and assessments, leading to decreased academic performance and increased dropout rates. In Saudi Arabia, approximately one-third of medical students reported difficulty comprehending English texts, with limited proficiency negatively influencing their exam results [10, 17]. Additionally, the use of foreign languages in teaching has been linked to feelings of inadequacy among students; many feel unprepared for clinical interactions due to insufficient language skills [19, 60]. This disconnect between the language of instruction and students’ native languages not only hampers their academic success but also exacerbates stress and anxiety related to performance evaluations [21, 34, 49].

For example, in Saudi Arabia, Almoallim et al. found that poor English proficiency was ranked as the second most significant difficulty faced by first-year medical students, with female students identifying it as their top challenge [19]. Similarly, Alqahtani reported that while Saudi nursing students had high academic achievement, their English language usage was low, particularly in speaking and writing, which significantly influenced their academic performance [21]. In Morocco, Abou Sahda et al. revealed that a third of medical students felt that French, the language of instruction, hindered their academic success, with 44% reporting increased learning time and 21% struggling to communicate with patients [61].

Moreover, a study in Sri Lanka found that English language proficiency was a significant predictor of academic performance among dental students, with weaker English skills correlating with lower GPAs [23]. In India, Amulya et al. reported that 88.5% of medical students could not comfortably communicate in the local language, leading to barriers in bedside teaching and learning [22]. These findings underscore the pressing need for educational reforms that accommodate native languages to enhance comprehension, participation, and success in medical education.

### Barriers in communication skills with patients

Language barriers significantly impact medical students’ communication skills with patients, often resulting in suboptimal care and patient outcomes (Table 1). Many studies reveal that students trained primarily in a foreign language struggle to effectively convey empathy, understand patient concerns, and conduct thorough medical histories in their native language [11, 29]. For instance, students from the United Arab Emirates reported feeling more comfortable and confident communicating with patients when using their native language, highlighting the challenges posed by English as a language of instruction [28, 32]. Moreover, the lack of proficiency in English among students has led to difficulties in patient interactions, with many expressing a desire for additional training in their native language to enhance their communication abilities [25, 60].

In Qatar, Diab et al. found that pharmacy students felt more confident performing OSCEs in Arabic, their native language, compared to English [25]. They also believed that using Arabic in OSCEs could improve patient care. Similarly, Alnahdi et al. reported that only 33.8% of medical students in Saudi Arabia felt confident taking medical histories in Arabic after being trained in English, with 68% recommending the addition of short Arabic history-taking courses to improve their skills [60].

In the United Arab Emirates, Hashim et al. found that medical students trained in English struggled with complex communication tasks, such as expressing empathy and eliciting patients’ expectations, during patient interactions [29]. This was further supported by another study, that reported that only 28% of medical students felt confident taking a patient history in Arabic after learning communication skills in English [11]. Similarly, Jabali’s study in Palestine found that half of the medical students preferred Arabic over English as the language of instruction, with many expressing concerns that using English might hinder their ability to communicate with patients after graduation [33]. These findings highlight the critical need for integrating native language instruction into medical curricula to improve communication skills, ensure better patient care, and foster a more empathetic healthcare environment.

## Discussion

This review highlights the profound challenges posed by language barriers in foreign-language-based medical education, particularly in countries where instruction is delivered in a non-native language. These barriers significantly affect academic performance and patient communication, emphasizing the need for reforms that balance the benefits of internationalization with the practical advantages of native-language instruction. Such reforms could improve educational outcomes and healthcare delivery while maintaining global competitiveness.

### The argument for internationalization vs. native language dependence

The debate between using English as the medium of instruction and relying on native languages is central to the future of medical education [3, 62]. On one hand, English is the dominant language of global medical research, scientific communication, and international collaboration. Adopting English as the medium of instruction allows students and professionals in non-English-speaking countries to access a vast repository of medical knowledge, participate in global conferences, and collaborate with international peers. For example, many students of the included studies, while faced challenges in English-medium instruction, acknowledged its importance for global scientific engagement.

On the other hand, the reliance on English in countries where it is not the native language creates significant barriers for students. Studies from the Arab world demonstrate that students often struggle with comprehension, retention, and application of medical knowledge when taught in English. Linguistic differences between L1 (Arabic) and L2 (English) further exacerbate these challenges. For instance, Kakar and Sarwari (2022) found that L1 (Farsi Dari) can both scaffold and interfere with L2 (English) communication [63]. While L1 proficiency helps generate ideas, improve self-esteem, and reduce anxiety, it also creates interference in pronunciation, grammar, and vocabulary. These findings align with the challenges faced by medical students in the Arab world, where Arabic (L1) often interferes with English (L2) medical terminology and communication. This disconnect not only hampers academic performance but also affects students’ ability to communicate effectively with patients in their native language, as seen in Hashim and Mirza studies [11, 29]. The preference for native language instruction is evident in studies like Jabali 2022, where 50% of Palestinian medical students favoured Arabic over English, citing better comprehension and patient communication [33].

A balanced approach that enhances English proficiency while incorporating native language instruction may offer the best of both worlds [3]. For instance, bilingual education programs, such as those combining Arabic and English in clinical training, have shown promise in bridging the gap between global standards and local needs [64, 65]. By teaching core medical subjects in the native language and providing supplemental English classes, students can achieve better comprehension of complex concepts while maintaining the ability to engage with international medical literature and practices.

### Academic and educational barriers

The studies reviewed highlight that foreign-language instruction, particularly in English, presents significant challenges to students’ academic performance. Overwhelming evidence from countries like Saudi Arabia and the United Arab Emirates shows that students often struggle to comprehend English-language textbooks and assessments, leading to suboptimal learning outcomes, increased stress, and higher dropout rates [10, 19]. These findings resonate with the need to reconsider language use in medical curricula, especially in non-English-speaking countries. While English proficiency is important for global scientific engagement, it should not come at the cost of students’ comprehension and success in their primary education [3].

Integrating native-language instruction into medical education would likely reduce these academic barriers. As demonstrated in the results, when students struggle with foreign-language comprehension, their academic performance suffers, resulting in increased stress and reduced retention of critical knowledge. A bilingual approach, where core subjects like anatomy, physiology, and pathology are taught in students’ native languages, can help ease their linguistic challenges while fostering better understanding and engagement with complex medical concepts [64, 65]. The incorporation of native-language training could thus lead to improved academic outcomes, ensuring that students are better prepared for clinical practice.

### Patient communication and cultural competence

The second major theme identified in the review is the impact of language barriers on students’ ability to communicate effectively with patients. Medical students trained in foreign languages often report difficulties in conveying empathy, gathering medical histories, and addressing patients’ concerns in their native languages. Students trained primarily in English, for example, may have a good grasp of medical terminology but find it challenging to apply this knowledge in their native language, which is crucial for building rapport and providing culturally sensitive care.

Many included studies highlighted that students were more comfortable and confident in-patient interactions when using their native language, illustrating how language can influence the quality of healthcare interactions. Medical education systems that rely solely on a foreign language for training inadvertently create a gap in effective communication between students and patients, leading to poorer patient outcomes [11]. Furthermore, this issue extends beyond language skills; it also involves cultural competence. In many regions, including the Middle East, healthcare delivery is deeply influenced by cultural norms that shape the way patients express concerns and interact with healthcare providers. The inability of students to effectively communicate in their native language can hinder their ability to engage with patients in culturally appropriate ways, ultimately impacting the quality of care [9]. To address these barriers, incorporating native language instruction in communication skills training is essential.

### Pedagogical implications and feasible solutions

The findings of this review have significant implications for medical education, particularly in countries where foreign languages are used as the medium of instruction. Based on the evidence, we propose actionable strategies and feasible solutions to address language barriers and improve educational outcomes. Adopting bilingual education, where students learn both in a foreign language and their native language, presents a feasible starting point in the transition towards fully native language-based medical education. A fully native language-based medical education does not imply abandoning English, which remains the leading language of global medical research and communication. Instead, it emphasizes the importance of learning English alongside the native language to ensure students are equipped for both local and international contexts. The studies reviewed suggest that bilingual programs, such as those combining Arabic and English in clinical training, offer a practical solution for addressing language barriers. These programs help students bridge the gap between their native language and the foreign language of instruction [64].

AI tools, particularly advanced translation technologies and large language models, can significantly support this transition to native-language education. AI-driven translation systems could enable students to translate any medical content—whether textbooks, research articles, case studies, or clinical guidelines—into their native language, thereby overcoming the limitations of foreign-language instruction [66]. This would allow students to access all learning materials in a language they are comfortable with, improving comprehension and facilitating deeper learning.

### Complexities in multilingual nations and the role of english-medium schools

The situation of language barriers in medical education becomes more complex in multilingual nations, where multiple languages coexist, and each region or state may have its own dominant language. For example, in countries like India, where there are 22 officially recognized languages and hundreds of dialects, the choice of language for medical education is not straightforward [67]. Patients in such settings often communicate in their regional languages, which may differ from the language of instruction in medical schools. This creates additional challenges for medical students, who must navigate multiple languages to effectively communicate with patients and deliver culturally sensitive care [22, 54].

Moreover, the prevalence of English-medium schools in many British Commonwealth nations, including India, adds another layer of complexity. In these schools, all subjects are taught in English, and students often become more proficient in English than in their native or regional languages. While this familiarity with English can facilitate the transition to English-based medical education, it may also lead to a disconnect between students and patients who primarily communicate in local languages. For instance, Amulya et al. found that 88.5% of medical students in India struggled to communicate comfortably in the local language during bedside teaching, despite being educated in English [22]. This highlights the need for medical curricula to address both the linguistic diversity of patients and the varying levels of language proficiency among students.

In such contexts, a one-size-fits-all approach to language in medical education is unlikely to be effective. Instead, tailored solutions that consider regional linguistic diversity and the prevalence of English-medium education are needed. For example, medical schools in multilingual nations could adopt a flexible approach, offering instruction in both English and the dominant regional language(s). This would allow students to develop proficiency in the languages most relevant to their clinical practice while maintaining the ability to engage with global medical knowledge. Additionally, communication skills training could be designed to address the specific linguistic needs of different patient populations, ensuring that students are well-prepared to provide effective and empathetic care in diverse settings.

### Policy implications

Policymakers have an important role in shaping educational reforms that address these language barriers. The successful incorporation of native-language medical education, as seen in several global initiatives, shows that it is possible to align with international medical standards while respecting local linguistic and cultural contexts [3]. Countries could adopt a gradual shift towards native-language curricula for foundational medical subjects, combined with ongoing English language support to maintain international competitiveness. A policy shift in this direction would not only improve students’ educational outcomes but also ensure that healthcare professionals are better equipped to deliver culturally sensitive care.

### Strengths and limitations of the study

This systematic review has several strengths, including its comprehensive search strategy across multiple databases (PubMed, Scopus, and Web of Science) and adherence to PRISMA guidelines, which ensured a rigorous and transparent review process. The inclusion of 49 studies involving over 14,500 students from diverse regions, particularly the Arab world, provides a robust evidence base for understanding the impact of language barriers in medical education. Theoretically, this study contributes to cognitive load theory and sociocultural learning frameworks by demonstrating how foreign-language instruction increases extraneous cognitive load and hinders culturally congruent patient care. Additionally, this is the first systematic review to comprehensively examine language barriers in medical education across countries that rely on foreign languages for instruction.

However, this review also has limitations. First, the majority of included studies were conducted in the Arab world, which may limit the generalizability of findings to other regions. Second, the heterogeneity in study designs (qualitative, quantitative, and mixed methods) and outcomes made it challenging to perform a meta-analysis, necessitating a narrative synthesis approach. The review focused on language barriers in medical education but did not explore the broader socio-political and historical factors that contribute to the reliance on foreign languages in many countries [5]. Despite these limitations, this review provides valuable insights into the challenges posed by language barriers in medical education and offers practical recommendations for addressing these issues. Future research should explore the long-term impact of bilingual education programs and the role of technology in bridging language gaps in medical education.

## Conclusion

Language barriers in foreign-language-based medical education significantly impede students’ academic performance and patient communication skills. This systematic review highlights the cognitive and emotional challenges students face when learning in a non-native language, as well as the disconnect between classroom training and real-world clinical interactions. Addressing these challenges through targeted reforms is crucial to enhancing the quality of medical education and ensuring effective healthcare delivery. To bridge these gaps, we propose a bilingual education model that integrates native languages into core medical curricula while maintaining English proficiency training. This approach balances the benefits of global scientific engagement with the practical advantages of local language proficiency. Additionally, leveraging AI-driven translation tools can provide real-time support for students, reducing barriers to accessing foreign-language resources. Future research should explore the long-term outcomes of bilingual programs and the efficacy of AI tools in bridging language gaps. By addressing these barriers, institutions can enhance educational equity and prepare healthcare professionals to meet the linguistic and cultural needs of diverse populations.

## Electronic supplementary material

Below is the link to the electronic supplementary material.

Supplementary Material 1

## Data Availability

The datasets used and analyzed during the current study are available from the corresponding author on reasonable request.

## Abbreviations

AI: Artificial Intelligence
EMI: English as a Medium of Instruction
GPA: Grade Point Average
L1: First Language (Native Language)
L2: Second Language (Foreign Language)
MCQ: Multiple Choice Question
MMAT: Mixed Methods Appraisal Tool
OSCE: Objective Structured Clinical Examination
PRISMA: Preferred Reporting Items for Systematic Reviews and Meta-Analyses
TOEFL: Test of English as a Foreign Language
