# Supplementary material for: Decolonizing medical education: a systematic review of educational language barriers in countries using foreign languages for instruction
Source: BMC Med Educ. 2025 May 13;25:701. doi: 10.1186/s12909-025-07251-2 (PMC12077016; doi:10.1186/s12909-025-07251-2)
Supplement: Supplementary file 1 — Supplementary Material 1 [file 12909_2025_7251_MOESM1_ESM.docx]

**Decolonizing Medical Education: A Systematic Review of Educational Language Barriers in Countries Using Foreign Languages for Instruction***(Supplementary file)*

**Table**: Quality assessment of the included studies

| ***Qualitative*** | | | | | | | |
| --- | --- | --- | --- | --- | --- | --- | --- |
| Study | Are there clear research questions? | Do the collected data allow to address the research questions? | Is the qualitative approach appropriate to answer the research question? | Are the qualitative data collection methods adequate to address the research question? | Are the findings adequately derived from the data? | Is the interpretation of results sufficiently substantiated by data? | Is there coherence between qualitative data sources, collection, analysis and interpretation? |
| Abdulghani 2014 | Yes | Can't tell | Yes | Yes | Yes | Yes | Yes |
| Chhabra 2022 | Yes | Yes | Yes | Yes | Yes | Yes | Can't tell |
| Khan 2004 | Yes | Yes | Yes | Yes | Yes | Yes | Yes |
| Khan 2019 | Yes | Yes | Yes | Yes | Yes | Can't tell | Can't tell |
| Matthews 2018 | Yes | Yes | Yes | Yes | Yes | Yes | Yes |
| McLean 2013 | Yes | Yes | Yes | Yes | Yes | Yes | Yes |
| Sheikh 2022 | Yes | Yes | Yes | Yes | Yes | Yes | Yes |
| ***Quantitative*** | | | | | | | |
| Study | Are there clear research questions? | Do the collected data allow to address the research questions? | Is the sampling strategy relevant to address the research question? | Is the sample representative of the target population? | Are the measurements appropriate? | Is the risk of nonresponse bias low? | Is the statistical analysis appropriate to answer the research question? |
| Abi Raad 2016 | Yes | Yes | Yes | Can't tell | Yes | Can't tell | Yes |
| Abu Sadah 2021 | Yes | Yes | Can't tell | Can't tell | Yes | Yes | Yes |
| Alhamami 2021 | Yes | Yes | Can't tell | Yes | Yes | Can't tell | Yes |
| Al-Mahmoud 2013 | Yes | Yes | Yes | Yes | Yes | Can't tell | Yes |
| Almoallim 2010 | Yes | Yes | Can't tell | Can't tell | Yes | Can't tell | Yes |
| Alnahdi 2021 | Yes | Yes | Yes | Yes | Yes | No | Yes |
| Alqahtani 2022 | Yes | Yes | Yes | Yes | Yes | No | Yes |
| Ameayou 2023 | Yes | Yes | Can't tell | Yes | Yes | Can't tell | Yes |
| Amulya 2021 | Yes | Yes | Yes | Yes | Yes | Yes | Yes |
| Ariyasinghe 2012 | Yes | Yes | Yes | Yes | Yes | Yes | Yes |
| Diab 2019 | Yes | Yes | Can't tell | Yes | Yes | Can't tell | Yes |
| Gazzaz 2023 | Yes | Yes | Yes | Yes | Yes | Can't tell | Yes |
| Hasan 2017 | Yes | Yes | Yes | Yes | Yes | Yes | Yes |
| Hassan 1995 | Yes | Yes | Yes | Yes | Yes | Yes | Yes |
| Hashim 2013 | Yes | Yes | Yes | Yes | Yes | Yes | Yes |
| Higgins-Opitz 2012 | Yes | Yes | Can't tell | Yes | Yes | Yes | Yes |
| Ismaiel 2023 | Yes | Yes | Yes | Yes | Yes | Yes | Yes |
| Jameel 2019 | Yes | Yes | Can't tell | Yes | Yes | Yes | Yes |
| Jha 2019 | Yes | Yes | Yes | Yes | Yes | Can't tell | Yes |
| Kaliyadan 2015 | Yes | Yes | Yes | Yes | Yes | Yes | Yes |
| Khallof 2019 | Yes | Yes | Yes | Yes | Yes | Yes | Yes |
| Lucas 1997 | Yes | Yes | Yes | Yes | Yes | Yes | Yes |
| Matthews 2018 | Yes | Yes | Yes | Yes | Yes | Yes | Yes |
| Mirza 2010 | Yes | Yes | Yes | Yes | Yes | Can't tell | Yes |
| Mpofu 1998 | Yes | Yes | Yes | Yes | Yes | Yes | Yes |
| Oducado 2020 | Yes | Yes | Can't tell | Yes | Yes | Can't tell | Yes |
| Olajuyin 2022 | Yes | Yes | Yes | Yes | Yes | Yes | Yes |
| Phisalprapa 2016 | Yes | Yes | Yes | Yes | Yes | Yes | Yes |
| Pun 2023 | Yes | Yes | Yes | Yes | Yes | Yes | Yes |
| Sabbour 2012 | Yes | Yes | Yes | Yes | Yes | Can't tell | Yes |
| Seneviratne 2019 | Yes | Yes | Yes | Yes | Yes | Yes | Yes |
| Shukaili 2023 | Yes | Yes | Can't tell | Yes | Yes | Yes | Yes |
| Singh 2011 | Yes | Yes | Yes | Yes | Yes | Yes | Yes |
| Stupart 2008 | Yes | Yes | Can't tell | Yes | Yes | Yes | Yes |
| Tantawi 2016 | Yes | Yes | Can't tell | Yes | Yes | Can't tell | Yes |
| Tayem 2020 | Yes | Yes | Yes | Yes | Yes | Can't tell | Yes |
| Tenney 2019 | Yes | Yes | Yes | Yes | Yes | Yes | Yes |
| ***Mixed methods*** | | | | | | | |
| Study | Are there clear research questions? | Do the collected data allow to address the research questions? | Is there an adequate rationale for using a mixed methods design to address the research question? | Are the different components of the study effectively integrated to answer the research question? | Are the outputs of the integration of qualitative and quantitative components adequately interpreted? | Are divergences and inconsistencies between quantitative and qualitative results adequately addressed? | Do the different components of the study adhere to the quality criteria of each tradition of the methods involved? |
| Ahmed 1988 | Yes | Yes | Yes | Can't tell | Yes | Yes | Yes |
| Eagleton 2015 | Yes | Yes | Yes | Yes | Yes | Yes | Yes |
| Jabali 2022 | Yes | Yes | Yes | Yes | Yes | Yes | Can't tell |
| Khan 2021 | Yes | Yes | Yes | Yes | Yes | Yes | Yes |
| Qadeer 2023 | Yes | Yes | Yes | Yes | Yes | Yes | Can't tell |
| Rabadi 2020 | Yes | Yes | Yes | Yes | Yes | Yes | Yes |
